# Supplementary material for: Complexome profiling of the Chlamydomonas psb28 mutant reveals TEF5 as an early PSII assembly factor
Source: Plant Cell. 2025 Mar 17;37(6):koaf055. doi: 10.1093/plcell/koaf055 (PMC12164590; doi:10.1093/plcell/koaf055)
Supplement: koaf055_Supplementary_Data [file koaf055_supplementary_data.zip › psb28_interactive.zip!/plots/intensity/qualityPlots/peptides/psbN_Cre-1.g2717031.t1.p1.html]

### Cre-1.g2717031.t1.p1

Trivial name: psbN  
SeqCoverage [median]: 25.00  
Mass: 5.21 kDa  
  
 

| Sequence | MissedCleavage | Mass [Da] | wtIntensity | muIntensity | Score |
| --- | --- | --- | --- | --- | --- |
| DPFEEHED | 0 | 1016.37 | 0.00 | 587.77 | 104.11 |
| KLRDPFEEHED | 2 | 1413.65 | 1092.89 | 1077.73 | 108.70 |
| LRDPFEEHED | 1 | 1285.56 | 22082.39 | 41795.97 | 152.27 |
|  |  |  | 23175.3 | 43461.5 |  |
